# Dry period heat stress induces microstructural changes in the lactating mammary gland

**DOI:** 10.1371/journal.pone.0222120

**Published:** 2019-09-19

**Authors:** Bethany Dado-Senn, Amy L. Skibiel, Thiago F. Fabris, Geoffrey E. Dahl, Jimena Laporta

**Affiliations:** Department of Animal Sciences, University of Florida, Gainesville, FL, United States of America; Tokat Gaziosmanpasa University, TURKEY

## Abstract

The bovine dry period is a non-lactating period between consecutive lactations characterized by mammary gland involution and redevelopment phases to replace senescent mammary epithelial cells with active cells primed for the next lactation. Dairy cows exposed to heat stress during the dry period experience milk yield reductions between 3–7.5 kg/d in the next lactation, partially attributed to processes associated with mammary cell growth and turnover during the dry period. However, the carry-over impact of dry period heat stress on mammary morphology during lactation has yet to be determined. In the current study, we hypothesized that exposure to heat stress during the dry period would alter alveolar microstructure and cellular turnover (i.e. proliferation and apoptosis) during lactation. Cows were either subjected to heat stress (HT, access to shade; *n* = 12) or cooling (CL, access to shade, fans, and soakers; *n* = 12) for a 46 d dry period. Upon calving, all cows were treated similarly with access to cooling for their entire lactation. Six cows per treatment were randomly selected for mammary gland biopsies at 14, 42, and 84 days in milk. Tissues were sectioned and stained for histological analysis. During lactation, HT cows produced 4 kg less colostrum and 3.7 kg less milk compared with CL cows. Lactating mammary gland microstructure was impacted after exposure to dry period heat stress; HT cows had fewer alveoli and a higher proportion of connective tissue in the mammary gland relative to CL cows, however alveolar area was similar between treatments. Rates of mammary epithelial cell proliferation and apoptosis were similar between treatment groups. This suggests that heat stress exposure during the dry period leads to reductions in milk yield that could be caused, in part, by a reduction in alveoli number in the lactating mammary gland but not to dynamic alterations in cellular turnover once lactation is established.

## Introduction

Heat stress is a costly and wide-spread concern to the dairy industry. Physiologically, heat stress is induced when a dairy cow is pushed above her thermoneutral zone at a temperature-humidity index (**THI**) of 68, forcing the cow to promote heat loss through sweating and panting and reduce heat production by limiting feed intake and decreasing milk production [1–3]. Rising global temperatures impair dairy cow productivity by reducing milk production, impairing reproductive success, and increasing morbidity and mortality. This leads to annual economic losses around $1 billion when lactating cows experience heat stress [4–7].

Even when not directly exposed to heat stress during lactation, a dairy cow can experience negative consequences when heat stress occurs during the dry period. We have previously shown that cows exposed to heat stress during the dry period experience decreases in milk production by 3 to 7.5 kg/d and increased incidences of health disorders in the next lactation [8–11]. Indeed, a recent economic analysis predicted that if cows are not provided heat stress abatement during the dry period, milk production losses could cost the US dairy industry an additional $810 million annually [12]. The dry period is a non-lactating state initiated six to eight weeks before parturition and is characterized by phases of mammary gland involution via programmed cell death (i.e. apoptosis) and redevelopment via cell division and growth (i.e. cellular proliferation) [13]. The purpose of this period is to promote mammary epithelial cell (**MEC**) turnover, where senescent epithelial cells are replaced with fully-functional cells in preparation for the next lactation [14]. Once lactation begins, the overall capacity of the mammary gland to synthesize and store milk is dependent on the number of MECs, which is regulated by rates of apoptosis and cellular proliferation, and the activity per cell [15].

Heat stress impacts cellular morphology and processes such as apoptosis and proliferation in the mammary gland. *In vitro* heat shock in connective and epithelial tissue results in a number of morphological alterations including structure and function of the plasma membrane, disruption of the Golgi complex, mitochondrial swelling, and alterations in cytoskeletal elements [16,17]. Specifically in mammary tissue, heat stress impairs MEC protein synthesis, alters organization of keratin and actin filaments, and downregulates genes involved in cellular growth and ductal branching while promoting expression of genes involved in apoptotic, phagocytic, and cell survival responses [18–23]. In addition, we have shown that heat stress experienced by the developing fetus in-utero (i.e. during late gestation, coinciding with the dry period) leads to reduced mammary alveolar area and MEC proliferation during these animals’ first lactation [24]. Dry period heat stress has been shown to impair MEC proliferation, thus potentially impairing the ability of the hyperthermic dry cow to successfully undergo the redevelopment phase in preparation for the next lactation [9,25].

While we understand some direct implications of heat stress on dry period mammary development in the dam and the long-lasting repercussions in the offspring, less is known about the carry-over effect of dry period heat stress on mammary morphology in the dam’s upcoming lactation. Thus, the objective of this study was to determine the impact of dry period heat stress on dairy cow mammary microstructure and cellular processes as they enter the next lactation. We hypothesized that dry period heat stress would alter alveolar architecture and impair MEC proliferation and apoptosis during lactation, thus reducing capacity for milk synthesis and storage which may further explain observed reductions in milk yield.

## Materials and methods

### Animals and treatments

#### Cows

This study was approved by the Institutional Animal Care and Use Committee at the University of Florida and was conducted from May to December 2016 at the University of Florida Dairy Unit (Hague, FL) using a herd of multiparous Holstein cows. Cows were dried off approximately 46 days before expected calving date according to UF Dairy Unit standard operating procedures and randomly assigned to one of two treatment groups for the duration of the dry period; heat stressed (**HT**; *n* = 12) or cooled (**CL**; *n* = 12). Mature-equivalent milk production in the previous lactation and parity were similar between treatments. For the entire dry period, both groups were housed in a shaded, sand-bedded free-stall barn; the CL group had access to water soakers and fans whereas the HT group did not. Fans ran continuously whereas water soakers were activated for 1.5 min in 6 min intervals when ambient temperature exceeded 21.1°C. Ambient temperature and relative humidity of the barn were recorded every 15 min for the entire dry period with Hobo Pro series Temp probes (Onset Computer Corp., Pocasset, MA). The temperature humidity index (**THI**) was calculated for the HT and CL areas of the barn based on the equation by Dikmen et al. [26]: THI = (1.8 x T + 32)–[(0.55–0.0055 x RH) x (1.8 x T– 26)], T = ambient temperature (°C) and RH = relative humidity (%). During the entire dry period, cows were fed a common close-up total mixed ration (**TMR**), formulated to the National Research Council recommendations (14.11% CP, 45.15% NDF, 0.68% Ca, 0.32% P, 0.12% Na, 1.06% Cl, 0.51% Mg, 0.32% S). Daily dry matter intake (**DMI**) of individual cows was measured from dry-off to calving and from calving up to 42 days in milk (**DIM**) using a Calan gate system (American Calan Inc., Northwood, NH). After calving, all cows were treated similarly, housed in shaded, free-stall barns with water soakers and fans and fed lactating cow TMR (17.54% CP, 30.38% NDF, 0.88% Ca, 0.44% P, 0.54% Na, 0.55% Cl, 0.42% Mg, 0.22% S). Cows were milked two times daily according to Dairy Unit standard operating procedure.

### Physiological measures and milk yield

During the dry period, rectal temperature was recorded twice daily at 0730 h and 1430 h, and respiration rate three times per week at 1400 h by counting the number of flank movements in one minute. Post-calving, rectal temperature and respiration rate were recorded every 7 days at 1430 h and 1400 h, respectively, until 84 DIM. Colostrum yield, daily milk yield, and cow body weights were obtained from AfiFarm Dairy Herd Management Software (Afimilk Ltd., Kibbutz, Afikim, Israel) from birth to 84 or 280 DIM (for weight and milk yield respectively). Gestation length was calculated by subtracting calving date from the artificial insemination date. Calf birth weight was recorded prior to first colostrum feeding.

### Mammary biopsies and tissue processing

Mammary biopsies were collected on 14, 42, and 84 DIM for a randomly selected subset of cows (*n* = 6 per treatment). Biopsies were collected according to the procedures from Farr et al. with slight modifications [27]. Briefly, cows were sedated via intravenous xylazine (Akom, Inc., Decatur, IL; 35 μl/kg BW). The biopsy site was selected, taking care to avoid major blood vessels, and sterilized by shaving and scrubbing with iodine and ethanol solutions. Tissue was collected in alternating rear right and left quarters of the gland between consecutive biopsies which were at least 28 d apart. Local anesthesia was provided through lidocaine (3 mL) administered subcutaneously at the biopsy site. A stainless-steel biopsy tool on a drill was inserted into a 3–4 cm incision in the mammary parenchyma and was activated to cut a core of mammary tissue. After collection, the biopsy incision was closed using sterile 18 mm Michel wound clips and sprayed with an antibacterial adhesive. Clips were removed after 3 days. The tissue collected was immediately washed in sterile saline, trimmed and sectioned, and stored overnight in cassettes in 4% paraformaldehyde at 4°C. The cassettes were subjected to a serial ethanol rinse (25%, 50%, 70% and 100%) for dehydration and returned to 70% ethanol for storage at 4°C. Following the standard procedures of the University of Florida Molecular Pathology Core, tissues were processed, paraffin embedded, sectioned at 5 μm, and affixed to slides. These sections were stained with hematoxylin and eosin (H&E), Masson’s trichrome, or further processed for immunohistochemistry (Ki67 and TUNEL). The H&E and Masson’s trichrome staining were conducted by the Molecular Pathology Core according to standard manufacturer protocol at standard times for animal tissue with histology grade xylene and graded ethanol series (Hematoxylin 7211, Clarifier1, Bluing, and Eosin Y Alcoholic; Thermo Fisher Scientific). These stained slides were used to identify, analyze, and quantify mammary tissue microstructure (i.e. alveoli area and number) and connective tissue area.

### Immunohistochemistry

#### Ki67 assay for cell proliferation

The Ki67 assay was performed to detect the number of proliferating cells (stained brown; positive) and non-proliferating cells (stained blue; negative) in a section of mammary tissue. The protocol for Ki67 staining was previously described in a companion study [24] using the UltraVision One Detection System: HRP Polymer/DAB Plus Chromogen kit (Thermo Fisher Scientific, Cat# TL-015-HDJ). Briefly, sections were deparaffinized and rehydrated. For antigen retrieval, slides were placed in coplin jars containing heated 1x citrate buffer in a 95°C water bath for 20 min, cooled for 10 min, then washed. Unless otherwise stated, all washes were performed in 1x tris-buffered saline with 0.1% Tween-20 (**TBSt**) and all incubations occurred at room temperature (~ 25°C). Sections were incubated with H_2_O_2_ for 15 min, washed, and incubated with UltraV block for 5 min. Slides were incubated for 30 min with primary antibody (Monoclonal Mouse Anti-Human Ki67 Antigen Clin MIB-1, Dako Agilent, Cat# M724029-2) previously diluted 1:100 in 1x TBSt. Upon washing, slides were incubated with Ultravision One HRP polymer for 30 min, washed again, then incubated with diluted DAB Plus chromogen for 5 min. Sections were washed with ddH_2_O and counterstained with hematoxylin for 1 min. Sections were dehydrated in an ethanol and xylene wash series and mounted with Permount (Thermo Fisher, Cat# SP15). Positive and negative controls were established using calf jejunum (high proliferative rates) and bovine mammary tissue at 14 DIM without primary antibody incubation, respectively.

#### Tdt dUTP nick-end labeling (TUNEL) assay for apoptosis

The TUNEL assay was conducted to quantify the number of cells undergoing apoptosis (stained brown; positive) and those not undergoing apoptosis (stained blue; negative) in a section of mammary tissue. The procedure followed was previously described in a companion paper [24] Briefly, we used the ApopTag Plus Peroxidase *in situ* Apoptosis Kit (Millipore, Cat# S7101) according to manufacturer’s instructions. Tissues were deparaffinized and rehydrated, and antigen retrieval was performed by incubating slides in 20 μg/ml proteinase K (Invitrogen, Cat# AM2542) for 8 min. Unless otherwise stated, incubations occurred at room temperature. Slides were washed in ddH_2_O then quenched in 3% H_2_O_2_ for 10 min. Slides were incubated in equilibration buffer for 10 min then incubated in a 1:3 working dilution of TdT enzyme in reaction buffer for 60 min at 37°C. Sections were washed in stop/wash buffer and 1x PBS followed by antibody incubation of anti-digoxigenin-peroxidase for 30 min and a DAB incubation for 8 min. Counterstaining was applied using methyl green stain (Sigma, Cat#198080), then slides were washed 3x. The sections were dehydrated in butanol and xylene washes and mounted with Permount (Thermo Fisher, Cat# SP15). Positive and negative controls were rat mammary tissue at 3–5 d involution (high apoptotic rates) and a section of bovine mammary tissue at 14 DIM incubated with TdT enzyme diluted in stop/wash solution instead of reaction buffer, respectively.

### Quantification of histological sections

The EVOS XL Core imaging system (Advanced Microscopy Group Bothell, WA) was used to capture images of H&E, Masson’s trichrome, Ki67, and TUNEL histological sections. ImageJ software was used for tissue quantification. The ImageJ Point Picker plugin was utilized to count alveoli number in H&E stained sections and the number of positive and negative cells in Ki67 and TUNEL sections. Area measurements for individual alveolar area (H&E) and connective tissue area (Masson’s trichrome) were calibrated to the image size and magnification (i.e. pixels to μm). Total tissue area for photomicrographs taken at 20x is 574,497 μm^2^ and at 40x is 96,820 μm^2^. Alveolar area was traced using the freehand tool, whereas connective tissue area was quantified by threshold measurement. Briefly, Masson’s trichrome images were converted to RGB stack color (black and white) and set at threshold of 55 for all sections. This converted connective tissue stain to a red color and all other tissue stain to white. The percentage area was calculated as the area of the red color as a proportion of the total image area.

For H&E stained sections, three photomicrographs were captured per section with a 10X objective for quantification of alveolar number and a 20X objective for alveoli area. The 10X magnification was chosen for alveolar number to ensure alveoli presence in each field. All photomicrographs captured were selected from random fields of view, taking care to avoid tissue edges. Alveoli were defined as structures with a lumen surrounded by a single layer of MEC, and alveolar area was quantified by measuring an individual alveoli’s luminal area excluding the surrounding MEC. Mammary ducts were not counted or measured. Care was taken to avoid all intralobular ducts and apparent interlobular ducts. Alveoli number and area were averaged across the three photomicrographs per section for each cow at each timepoint for statistical analysis.

Sections from Masson’s trichrome staining were taken at 20X magnification with three randomly captured photomicrographs per section to measure connective tissue area. Connective tissue was defined as tissue stained blue, whereas all other remaining tissue (i.e. alveolar cells, ductal epithelial cells, myoepithelial cells, immune cells, adipocytes, endothelial cells, and red blood cells) was stained pink or dark purple as a covariate. The remaining tissue area was not calculated. Connective tissue area was averaged across the three photomicrographs per section for each cow at each timepoint for statistical analysis.

Histological sections from Ki67 and TUNEL assays were captured with three photomicrographs taken randomly per section at a 40X objective. In each capture, every cell was counted and defined as either positive (stained brown) or negative (stained blue). Cells were classified as either MEC or stromal cell. Mammary epithelial cells encompassed alveolar cells, ductal epithelial cells, and myoepithelial cells, whereas stromal cells included cells in the surrounding epithelium such as fibroblasts, immune cells, adipocytes, endothelial cells, and red blood cells. Results are expressed as proportion of positive cells per section. To calculate the proportion of cells either proliferating (Ki67) or undergoing apoptosis (TUNEL), the total number of positive cells per section were divided by the total number of cells per section (positive and negative) for each cell type (MEC or stromal cells).

### Statistical analyses

Statistical analysis was conducted in SAS v. 9.4 (SAS Institute, Cary, NC) with all data tested for normality. PROC GLM was used for one-way ANOVA analysis of colostrum yield, gestation length, and calf birth weight with calf sex as a covariate and a sex by treatment interaction was included for calf weight. Cell proliferation or apoptosis was analyzed by generalized linear mixed models using PROC GLIMMIX with a binomial distribution and logit link function. Fixed effects included treatment, DIM, and their interaction, and cow ID within treatment as random effect. Correlation between alveoli number and MEC number was assessed using Pearson’s correlation (PROC CORR). All other data were analyzed by generalized linear mixed models using PROC MIXED with fixed effects of treatment, DIM (repeated measures), and their interaction. The first-order autoregressive covariance structure (AR(1)) was used as the covariate structure. Significance was declared at *P* ≤ 0.05 and tendency was declared at 0.05 < *P* ≤ 0.10. Data are presented as least squares means (LSM) ± standard error (SE) unless otherwise stated. The minimal data set can be found in S1 Table.

## Results

### Physiological measures and milk yield

During the dry period, THI was similar between pens (74.6 vs. 74.4 ± 0.3 for HT vs. CL respectively), yet respiration rate (69.5 vs. 53.8 ± 2.1, *P* < 0.01) and rectal temperature in the morning (38.56 vs. 38.35 ± 0.03, *P* < 0.01) and afternoon (39.09 vs. 38.74 ± 0.03, *P* < 0.01) were higher for the HT cows relative to CL cows across the duration of the dry period, indicating abatement of heat stress in the cooled group during the dry period was successful (Table 1). Respiration rate for both groups increased as the dry period progressed (*P* = 0.02). Dry matter intake was not different between treatments during the dry period (11.91 vs 9.83 ± 1.16, *P* = 0.24; Table 1).

**Table 1:** Physiological parameters, colostrum and milk yield. Multiparous Holstein cows were exposed to dry period heat stress (HT, *n* = 12) or cooling (CL, *n* = 12) for ~46 d pre-calving. Measurements occurred both during the dry period (when treatment was applied) and during lactation (after treatment exposure). Data are presented as LSM± SE.

	Dry Period (pre-calving)		Lactation (post-calving)		
Variable	HT	CL	HT	CL	*P-* value^1^
Rectal temperature AM (°C)	38.56 ± 0.03	38.35 ± 0.03	-	-	**<0.01**
Rectal temperature PM (°C)	39.09 ± 0.03	38.74 ± 0.03	-	-	**<0.01**
Respiration rate (bpm*)	69.52 ± 2.04	53.75 ± 2.12	-	-	**<0.01**
Dry matter intake (kg/d)	11.91 ± 1.16	9.83 ± 1.16	-	-	0.24
Rectal temperature PM (°C)	-	-	38.76 ± 0.05	38.66 ± 0.05	0.52
Respiration rate (bpm*)	-	-	57.11 ± 1.38	58.73 ± 1.45	0.43
Dry matter intake (kg/d)	-	-	17.97 ± 0.81	16.81 ± 0.80	0.34
Calf birth weight (kg)	-	-	37.56 ± 1.22	44.39 ± 1.27	**<0.01**
Gestation length (d)	-	-	270.7 ± 1.78	277.4 ± 1.65	**0.01**
Body weight (birth, kg)	-	-	715.54 ± 19.81	708.88 ± 19.81	**0.81**
Body weight (lactation, kg)	-	-	648.86 ± 9.14	675.99 ± 9.90	**0.06**
Colostrum yield (kg)	-	-	3.72 ± 0.71	7.79 ± 0.87	**<0.01**
Milk yield (kg/d)	-	-	31.15 ± 0.48	34.85 ± 0.53	**<0.01**

^1^ P-value is for the treatment effect. Bold-faced font indicates significant difference at *P* < 0.05. There was an interaction between treatment and DIM on respiration rate in the lactating cows (*P* = 0.02). All other interactions were not significant. As lactation progressed, rectal temperature and body weight decreased (*P* < 0.01, *P* = 0.06).

*bpm = breaths/minute.

Cows exposed to heat stress during the dry period had a shorter gestation length and gave birth to lower birthweight calves than did cows provided with active cooling during the dry period (*P* = 0.43, Table 1). There was no difference between treatment groups in body weight at calving (*P* = 0.81), but HT cows tended to weigh less over 84 DIM (*P* = 0.06, Table 1). Regardless of treatment, body weight also tended to change across 84 DIM, but there was no treatment by time interaction (*P*(DIM) = 0.06, *P*(INT) = 0.20). After calving, when both groups of cows were provided with active cooling, rectal temperature, respiration rates, and DMI were similar between treatment groups and averaged 38.71° C, 58 bpm, and 17.4 kg/d (*P* > 0.34, Table 1). As lactation progressed, rectal temperature of the cows decreased (*P* < 0.01). There was an interaction between DIM and treatment on respiration rate for the lactating cows (*P* = 0.02); whereby respiration rate was significantly higher at 7 DIM in HT cows but was higher in CL cows at 28 DIM.

**Fig 1:**
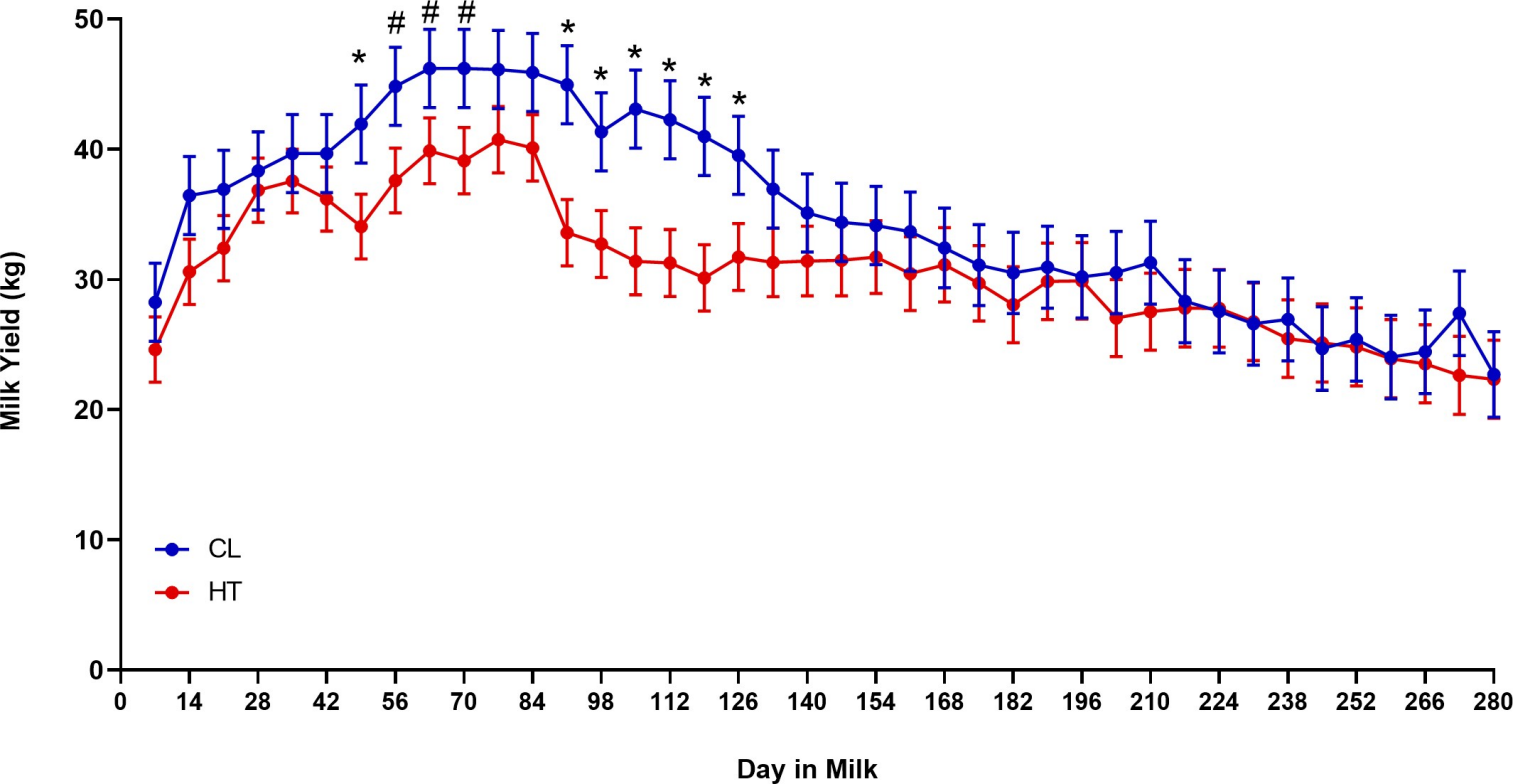
Weekly milk yield through 280 days in milk (DIM). Multiparous Holstein cows were heat stressed (HT, access to shade only, *n* = 12) or cooled (CL, access to shade, fans, and water soakers, *n* = 12) during their previous dry period (~46 d pre-calving). Milk yield was lower in HT compared to CL dams (*P* < 0.01). * indicates *P* ≤ 0.05 and # indicates 0.10 ≤ *P* > 0.05. All data are presented as LSM ± SE.

Cows that were cooled during the dry period produced 4 kg more colostrum and 3.7 kg/day more milk than HT cows across 280 DIM (Table 1, *P* < 0.01). Milk yield changed over the course of lactation for both groups (*P* < 0.01), but there was no interaction between DIM and treatment (*P* = 0.76, **Fig 1**).

### Mammary gland microstructure

**Fig 2:**
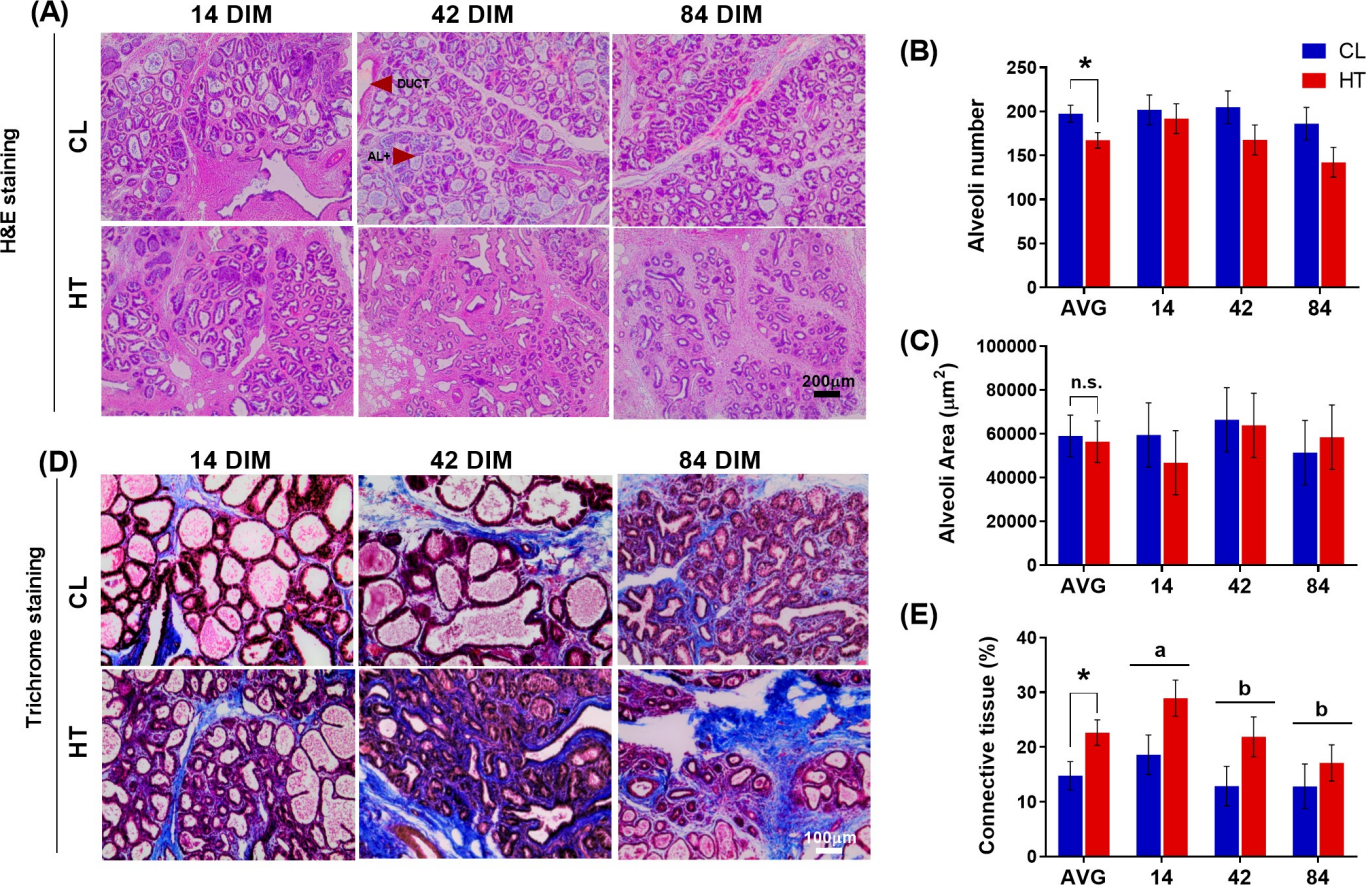
Histological evaluation of the lactating mammary gland. Multiparous Holstein cows were heat stressed (HT, access to shade only, *n* = 6) or cooled (CL, access to shade, fans, and water soakers, *n* = 6) during the entire dry period (~46 d pre-calving). Mammary biopsies were collected at 14, 42, and 84 days in milk (DIM). (A) Hematoxylin and eosin (H&E) stained mammary tissue at 10X, (B) number of alveoli in the mammary gland of CL and HT cows, (C) area of alveoli in mammary gland of CL and HT cows. (D) Masson’s trichrome stained mammary tissue at 20X, (E) proportion of connective tissue in mammary gland of CL and HT cows. Alveoli number was lower in HT compared to CL dams (*P* = 0.04). Alveoli area was not different between treatments (*P* = 0.84). The proportion of connective tissue was higher in HT compared to CL dams (*P* = 0.05) and higher at 14 DIM compared to 42 and 84 DIM (*P* = 0.04). No interactions between treatment and DIM were observed. All data are presented as LSM ± SE. Disparate letters indicate a significant difference over time (i.e. 14 DIM vs. 42 DIM, 14 DIM vs. 84 DIM, or 42 DIM vs. 84 DIM). An asterisk indicates a significant difference and n.s. indicates a non-significant difference between treatments. AL = alveoli, DUCT = mammary duct. Scale bar is 200 μm for H&E staining and 100 μm for Masson’s trichrome staining.

There were fewer alveoli in the mammary gland of HT cows compared to CL cows (167.3 vs. 197.7 ± 9.3, *P* = 0.04; **Fig 2A and 2B**) across 84 DIM, but individual alveoli area was similar between treatment groups (56345 vs. 59057 ± 9488 μm^2^, for HT vs. CL respectively, *P* = 0.84; **Fig 2C**). There were no significant interactions between DIM and treatment for alveoli area and number (*P* > 0.59). Alveoli number was positively correlated with the number of MEC (*r* = 0.89, *P* < 0.001). Cows heat-stressed during the dry period had a significantly higher proportion of mammary connective tissue during lactation relative to cows cooled during the dry period (22.7 vs. 14.8 ± 2.5% respectively, *P* = 0.05; **Fig 2D and 2E**). There was also a DIM effect whereby mammary tissue at 14 DIM had a greater percentage of connective tissue compared with 42 and 84 DIM (23.8 vs. 17.4 vs. 15.0 ± 2.5%, respectively, *P* = 0.04). No interaction between treatment and DIM was observed (*P* = 0.63).

### Cell proliferation and apoptosis

**Fig 3:**
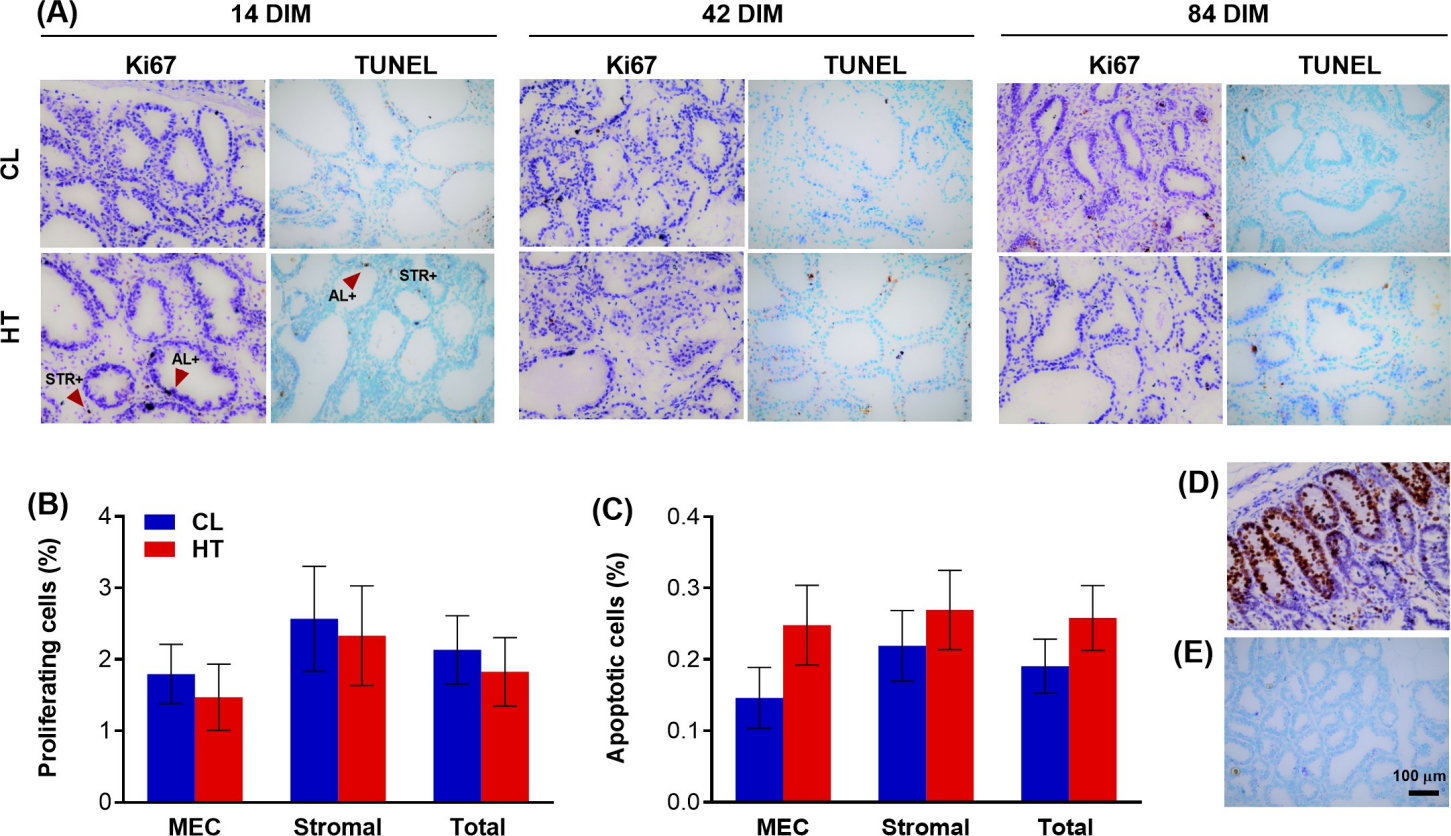
Mammary immunohistochemistry of proliferating and apoptotic cells in lactating Holstein cows. Multiparous cows were heat stressed (HT, access to shade only, *n* = 6) or cooled (CL, access to shade, fans, and water soakers, *n* = 6) during the entire dry period (~46 d pre-calving). Mammary biopsies were collected at 14, 42, and 84 days in milk (DIM). (A) Ki67 assay for cell proliferation and Tdt dUTP nick-end labeling (TUNEL) assay for apoptosis in mammary tissue at 40X, (B) proportion of proliferating cells in MEC, stromal, and total (MEC + stromal) mammary cells, and (C) proportion of apoptotic cells in MEC, stroma, and total mammary cells. Positive controls for (D) Ki67 (jejunum tissue from a 2-day old calf) and (E) TUNEL assays (rat mammary tissue at 3–5 d involution). Neither percentage of proliferating cells (*P* > 0.55) nor percentage of apoptotic cells (*P* > 0.18) was different between treatment groups. However, there was a significant DIM effect where the proportion of proliferating MEC and total cells was lower at 14 DIM compared to 84 DIM (*P* ≤ 0.04). All data are presented as LSM ± SE. Red arrows indicate positive cells (stained brown). AL = alveoli lumen, STR = stroma. Scale bar is 100 μm.

There was no difference between CL and HT dry period treatments in proportion of total proliferating cells (1.8 vs. 2.1 ± 0.5%, for HT vs. CL respectively, *P* = 0.55; **Fig 3A and 3B**) or proportion of MEC or stromal proliferating cells (*P* > 0.62) during lactation at 14, 42, or 84 DIM. There was a significant DIM effect where the percent of proliferating MEC and total cells (MEC + stromal) was decreased at 14 DIM compared to 84 DIM (MEC: 0.8 vs. 3.3 ± 0.6%, *P* = 0.02; Total cells: 1.3 vs. 3.2 ± 0.6%, *P* = 0.04). Exposure to heat-stress during the dry period did not impact the proportion of total apoptotic cells (0.26 vs. 0.19 ± 0.05%, for HT vs. CL respectively, *P* = 0.28; **Fig 3A and 3C**) or proportion of MEC or stromal apoptotic cells (*P* > 0.18) during lactation at 14, 42, or 84 DIM. There were no treatment by time interactions for cell proliferation or apoptosis (*P* > 0.35).

## Discussion

Dry period heat stress has been shown to cause milk yield losses in the subsequent lactation by our group and others [8,9,28–30], and is associated with lower MEC proliferation in the dry period [9]. However, less is understood about the carry-over effect of dry period heat stress on mammary morphology during the subsequent lactating period. This study describes the alterations to dairy cow mammary microstructure and cellular processes in the lactating mammary gland after heat stress exposure during the regenerative involution of the dry period. During the dry period, rectal temperature and respiration rate were elevated in the HT cows relative to CL cows despite a similar THI between the barns, indicating achievement of heat stress abatement for the CL group, consistent with previous studies [8,9,31]. After calving when all cows were provided heat stress abatement, previously CL and HT cows had similar rectal temperatures and respiration rates that fell within the thermoneutral ranges and were lower than those observed during the dry period for the HT cows. This indicates that upon initiation of lactation, when both groups were cooled, neither group was experiencing severe levels of heat stress [32]. Thus, we assume any differences between groups in terms of mammary morphology or cellular processes during lactation can be attributed to heat stress exposure during the dry period.

Cows exposed to heat stress during the dry period gave birth an average 7 days earlier and to lighter calves than CL cows, corroborating many studies of dry period heat stress in dairy cattle [33–35]. In the lactation following exposure to heat stress, HT cows produced 4 kg less colostrum and 3.7 kg/d less milk. This milk yield difference is within the reported range of a 3 to 7.5 kg/d difference in milk yield between multiparous cows that were either exposed to heat stress or provided fans and water soakers during the dry period [8–10,36]. As expected, milk yield changed across the lactation period. Contrasting the shape of the lactation curves of CL and HT cows, it is evident that there is increased milk production in cows that are CL during the dry period.

Both groups exhibit an increase in milk production that peaked around 84 DIM, which is typical for dairy cows in similar environmental conditions [37], however the increase in milk yield is greater in cows that were CL during the dry period. Biopsy collection timepoints of 14, 42, and 84 DIM occur before or at peak milk yield, thus capturing changes in mammary morphology and cellular processes during early and peak lactation, before the decline in lactation [15]. Further investigation of the impact of dry period heat stress on mammary histology during late lactation should be conducted, as milk yields for both treatment groups were similar at that time.

As previously shown by our group [9,36], cows under dry period heat stress consume approximately 1 kg/d less dry matter compared to those that were provided with heat abatement, although in this study it is not statistically different. Importantly, after calving, when both groups of cows are provided heat abatement, DMI was not different. This indicates that differences observed in milk production cannot be attributed to the impact of heat stress on reduced intakes and energy balance during lactation. Therefore, alterations in mammary morphology are likely to play a primary role in milk losses.

Alveoli number, but not individual alveoli area, was significantly lower during lactation in mammary tissue of cows exposed to dry period HT compared with CL cows. This is contradictory to a companion study of in-utero heat-stressed heifers that found a reduction in alveolar area, but not alveolar number, in the first lactation of heifers exposed to heat stress in-utero [24]. Discrepancies between these two studies may be attributed to the differences in timing of heat stress exposure, i.e. during the dry period immediately prior to lactation vs. during in-utero development nearly two years before lactation began. Indeed, the two responses may differ as the effect of in utero heat stress persists across multiple lactations whereas the impact of dry period heat stress on a mature cow to a second lactation is unclear [38]. Alternatively, differences may be explained by the biological influence of heat stress exposure, as dry period heat stress can lead to direct impacts on MEC cell death and proliferation while in-utero heat stress may alter fetal mammogenesis, methylation profile, or early stem cell development within the mammary gland [9,18,24,39]. Additionally, parity may impact these results, as primiparous mammals can experience improved MEC survival, altered MEC differentiation, and reduced alveolar size and density during lactation when compared to their multiparous counterparts [40–42].

Regardless of the foregoing differences, reductions in alveolar number and area can have negative consequences on overall luminal area and milk secretion and storage in the mammary gland, potentially leading to the losses in milk production evident in both studies [24,43]. As expected, alveoli number was highly correlated with MEC number, because alveoli are luminal structures surrounded by a single layer of MEC. Both MEC number and activity regulate the capacity of the mammary gland to synthesize and store milk, with cellular activity being the main driver of milk yields during early lactation in dairy cattle [44]. While we do not have a measure of activity per MEC in the current study, it is logical to predict that a significant reduction in alveolar number during early lactation would also prevent the overall activity and functionality of the mammary gland to reach its full synthetic capacity. In addition, secreted milk is stored in the alveoli and ducts of the gland until milk ejection; thus, mammary storage capacity is in part dependent on the number of alveoli [45].

Dry period HT cows had a significantly higher percentage of mammary connective tissue during lactation compared with cooled cows. Proportions of mammary epithelial tissue and connective tissue are inextricably linked; as the percentage of connective tissue increases, alveolar luminal area decreases and vice versa [46]. It is possible that the reduced parenchymal growth caused by dry period heat stress allows the existing connective tissue to persist resulting in the greater proportion observed during lactation. Consequently, this shift in tissue types may impact the overall secretory capacity of the gland. Consistent with our heat stress model, other stressors related to intramammary infection have been shown to reduce epithelial area while increasing stromal area during lactation, potentially leading to impaired milk production [47,48]. Traditionally, the secretory mammary epithelium is given primary consideration when analyzing the impact of heat stress on cellular morphology and production phenotype. However, this epithelium is closely linked to the stroma through the basement membrane extracellular matrix. Therefore, other supportive cell types such as fibroblasts and adipocytes may play crucial roles in mammary gland remodeling during the dry period and maintaining milk synthesis during lactation, particularly under heat stress [46,49]. Additional research is needed to understand connective tissue dynamics under dry period heat stress.

Dry period heat stress did not influence the proportion of proliferating cells nor the proportion of apoptotic cells during the first 84 DIM. Cellular apoptosis and proliferation can be greatly impacted by heat stress. *In vitro* heat shock initiates cell death of damaged MECs through apoptosis, impairs MEC cellular proliferation, and alters the cellular cytoskeleton through impaired protein synthesis to promote thermotolerance and protection of MECs [21–23]. Heat stress leads to downregulated expression of genes related to cellular growth and ductal branching and upregulated expression of genes involved in apoptotic, phagocytic, and cell survival responses [18–20,23]. However, in the current study, cows were directly exposed to treatment of heat stress or heat stress abatement only during the dry period, so lack of direct heat stress exposure later during lactation may reduce the likelihood of altered rates MEC cell death or proliferation. Indeed, Tao et al. (2011) demonstrated that dry period heat-stressed cows experienced impaired MEC proliferation during the late dry period that did not continue into lactation [9]. These same authors also found no differences in apoptotic rates during the dry period or lactation, evaluated at 2 and 20 DIM, consistent with the current study which expanded the sampling dates up to 84 DIM. However, other cell death processes, like autophagy, were impacted by dry period heat stress particularly during the early dry period [25]. Further investigation of these processes during lactation is warranted In addition, proliferative and apoptotic rates during early and peak lactation are not as dynamic as in the preceding dry period; thus may not be as greatly impacted by dry period heat stress [13,44,46]. Proliferation of MECs remains relatively low across lactation with a tendency for reduced rates during early lactation (i.e. 14 DIM) [44]. Apoptotic rates exceed rates of cellular proliferation, but do not drive lactation persistency until later in lactation when cellular apoptosis leads to an observed decline in mammary cell number [44]. In a companion study, in-utero heat stress did not impact rates of apoptosis but tended to impair MEC proliferation when these heifers reached their first lactation. Again, discrepancies between these two studies may be attributed to the differences in the biology behind the timing of heat stress exposure or parity, as primiparous cows exhibit differences in MEC differentiation during lactation [41,42].

After heat stress insult across the duration of the dry period, dairy cows experience reductions in milk yield that appears to be influenced, in part, by a reduction in alveoli number and an increase in connective tissue but not changes in cellular turnover during lactation. This is consistent with the shape of the lactation curves between treatment groups–reduced yield at the start of lactation but no differences in persistency across 35 weeks in milk. In the present study due to timing of mammary biopsies, we cannot determine if this alteration in alveolar and connective tissue architecture originates during the dry period or upon establishment of lactation around 14 DIM. However, alveoli area and number were consistent across the lactation period indicating that epithelial structure during lactation does not change dramatically. Connective tissue percentage was higher at 14 DIM compared to 42 and 84 DIM, but this may be a function of improved invagination of epithelial tissue outside of the effects of heat stress. Further, past research from our group suggests that direct exposure of dry period heat stress influences key processes in mammary gland development including MEC proliferation during the late dry period and ductal branching across the dry period, thus perhaps leading to a reduction in alveolar function and number before a cow enters the next lactation [9,18]. Additional examination of mammary histology across the entire duration of the dry period must be conducted to investigate the temporal relationship between dry period heat stress and alteration in mammary epithelial and stromal portions across both the dry period and lactation.

In conclusion, our data demonstrate altered morphology of the lactating mammary gland after dry period heat stress exposure, which may be associated with the decrease in milk production observed. Further studies are needed to elucidate the temporal relationship of this dry period heat stress on the initiation and establishment of impaired mammary microstructure and cellular processes and to determine the mechanisms by which alterations in alveoli architecture affect production phenotypes.

## Supporting information

S1 Table Minimal data set. Excel Masterfile with minimal data set for (a) milk yield and milk components, (b) vitals (respiration rate, rectal temperature) and growth, (c) mammary gland H&E, and Masons trichrome, (d) mammary gland proliferation and apoptosis (Ki67 and TUNEL).
(XLSX)
